# Association of Radiomics and Pericarotid Adipose Tissue Characteristics with Systemic Inflammation in Patients Undergoing Carotid Endarterectomy

**DOI:** 10.3390/jcm14238342

**Published:** 2025-11-24

**Authors:** Diogo Santos-Teixeira, Piotr Myrcha, Vasco Trigo, Hugo Ribeiro, João Barbosa-Breda, Marina Dias-Neto, João Rocha-Neves, Peter Gloviczki

**Affiliations:** 1 Faculty of Medicine, University of Porto, 4200-319 Porto, Portugal; 2 1st Chair and Department of General and Vascular Surgery, Faculty of Medicine, Medical University of Warsaw, 02-091 Warsaw, Poland; piotrmyr@poczta.fm; 3 Department of General, Vascular and Oncological Surgery, Masovian Brodnowski Hospital, 03-242 Warsaw, Poland; 4 Department of Biomedicine, Unit of Anatomy, Faculty of Medicine, University of Porto, 4200-319 Porto, Portugal; trigo.vasco@gmail.com; 5 RISE@Health, Departamento de Biomedicina, Faculty of Medicine, University of Porto, Alameda Prof. Hernâni Monterio, 4200-319 Porto, Portugal; hribeiroff@gmail.com (H.R.); joaorochaneves@hotmail.com (J.R.-N.); 6 Community Palliative Care Support Team Gaia, 4400-129 Vila Nova de Gaia, Portugal; 7 Departamento of Medicina da Comunidade, Informação e Decisão em Saúde, Faculty of Medicine, University of Porto, 4200-319 Porto, Portugal; 8 Centre for Innovative Biomedicine and Biotechnology, 3000-548 Coimbra, Portugal; 9 RISE@Health, Department of Surgery and Physiology, Faculty of Medicine, University of Porto, 4200-319 Porto, Portugal; joao_breda@hotmail.com; 10 Department of Ophthalmology, Centro Hospitalar e Universitário São João, 4200-319 Porto, Portugal; 11 Research Group Ophthalmology, Department of Neurosciences, KULeuven, 3000 Leuven, Belgium; 12 Angiology and Vascular Surgery, Unidade Local de Saúde de São João, 4200-319 Porto, Portugal; marina_f_neto@hotmail.com; 13 Surgery and Physiology, Faculty of Medicine, University of Porto, 4200-319 Porto, Portugal; 14 Department of Vascular Surgery, Unidade Local de Saúde do Alto Ave, EPE, 4835-044 Guimarães, Portugal; 15 Division of Vascular and Endovascular Surgery, Gonda Vascular Center, Mayo Clinic, Rochester, MN 55901, USA; gloviczki.peter@mayo.edu; 16 Faculty of Medicine, Semmelweis University, 1085 Budapest, Hungary

**Keywords:** vascular imaging, carotid stenosis, biomarkers, neutrophil-to-lymphocyte ratio, red cell distribution width, Hounsfield units

## Abstract

**Background:** Pericarotid adipose tissue (PCAT) characteristics and systemic inflammation may play an important role in carotid endarterectomy (CEA) outcomes. This study explores the association between PCAT Hounsfield Unit (HU) ranges, radiomic features, and systemic inflammatory markers in patients undergoing carotid endarterectomy (CEA). **Methods:** Twenty patients undergoing CEA were included in this cross-sectional study. PCAT was analyzed using preoperative computed tomography angiography (CTA) images, with regions of interest defined around the carotid arteries. PCAT was categorized into three HU ranges: −190 to −120, −119 to −70, and −69 to −30. Radiomics features were extracted using PyRadiomics. The primary outcome was the correlation of PCAT imaging with preoperative neutrophil-to-lymphocyte ratios (NLRs). The secondary outcome was the association of PCAT imaging with the red cell distribution width (RDW-CV). Linear regression was used to evaluate associations between PCAT characteristics and inflammatory markers. **Results:** Distinct HU ranges in PCAT imaging showed strong correlations with the preoperative NLR. The −190 to −120 HU range demonstrated a negative association (β = −3.809, *p* < 0.001), whereas the −119 to −70 HU range showed a positive correlation (β = 3.814, *p* < 0.001). PCAT uniformity was positively associated with RDW-CV (β = 0.494, *p* = 0.027). Other radiomics features, such as contrast, showed trends but did not reach statistical significance. A larger outer area of PCAT was inversely associated with the NLR (β = −0.677, *p* < 0.001). **Conclusions:** Specific PCAT HU ranges and radiomics features are significantly associated with systemic inflammatory markers in CEA patients. These findings suggest that HU-based segmentation and radiomics analysis of PCAT may offer valuable insights into the relationship between local adipose tissue characteristics and systemic inflammation.

## 1. Introduction

Carotid endarterectomy (CEA) is a preventive surgical procedure aimed at reducing the risk of stroke by removing atherosclerotic plaques from the carotid arteries. The role of systemic inflammation in the atherosclerosis progression and its impact on postoperative outcomes is increasingly recognized [1,2]. Emerging evidence indicates that inflammatory markers such as the neutrophil-to-lymphocyte ratio (NLR) and red cell distribution width (RDW) are reliable predictors of cardiovascular events [3,4].

Pericarotid adipose tissue (PCAT), the adipose tissue surrounding the carotid artery, has garnered attention for its significant role in vascular health [5]. As a biologically active tissue, PCAT secretes pro-inflammatory cytokines and adipokines that may contribute to plaque instability and elevate the risk of thromboembolic events [6]. Characterizing PCAT via imaging-particularly its distribution and attenuation values-offers novel insights into the interaction between local inflammation and systemic outcomes [5,7].

Radiomics has emerged as a powerful tool in vascular risk assessment by extracting quantitative biomarkers from medical imaging [7,8]. By detecting subtle patterns in CT, MRI, and ultrasound scans, radiomics aids in predicting progression of atherosclerosis, the risk of aneurysm rupture, and other vascular complications, supporting early diagnosis and personalized treatment strategies [7,9,10].

This study aims to evaluate the association between PCAT characteristics and systemic inflammatory markers, focusing on preoperative NLR and RDW values in patients undergoing CEA. Understanding this relationship could have significant implications for surgical risk stratification and outcomes [11].

## 2. Methods

### 2.1. Study Sample and Data Source

For this study, patients were recruited from a tertiary care referral centre where they underwent CEA under regional anaesthesia (RA) for the treatment of carotid stenosis, with patch angioplasty. The study included patients admitted between January 2022 and December 2022, with data collected from a prospective institutional database.

All patients underwent evaluation by a vascular surgeon and an anesthesiologist before surgery. For those with symptomatic stenosis, a neurologist also conducted an assessment. Patients were excluded if they underwent concurrent cardiac surgery, were unwilling to receive regional anaesthesia, or lacked a preoperative CT scan. All eligible patients within the study period were consecutively included; no sampling weights or complex survey methods were used.

A preoperative CT scan was obtained within two months of the scheduled surgery. Demographic data and comorbidities were recorded preoperatively. Postoperatively, all patients remained on optimal medical therapy, including single antiplatelet therapy and statins, with no specified end date. CEA under RA represents the standard of care at this center.

This study was conducted in accordance with the Strengthening the Reporting of Observational Studies in Epidemiology (STROBE) guidelines [12].

The study protocol adheres to the Declaration of Helsinki and complies with the European Union General Data Protection Regulation. Written informed consent was obtained from all participants after a thorough explanation of the study’s objectives, procedures, risk, and potential benefits. The study was approved by the institution’s ethics committee (Approval ID: 163-21 and 148-16) and is registered on ClinicalTrials.gov (Identifier: NCT05623293).

### 2.2. Definitions

According to current guidelines of the European Society for Vascular Surgery [13]. CS was classified as symptomatic or asymptomatic. This assessment was performed using either Doppler ultrasound, based on velocimetric criteria, or CT angiography using North American Symptomatic Carotid Endarterectomy Trial (NASCET) criteria [14].

TIA and stroke were defined using time-based criteria:

- TIA was defined as an episode of focal brain, retinal, or spinal cord dysfunction lasting less than 24 h.
- Stroke was defined as a sudden onset focal neurological dysfunction with symptoms lasting more than 24 h [13].

The cause of death was determined using the Death Certificate Information System (SICO™—Portuguese National Health Service—SPMS), the official mortality registry in Portugal.

Chronic kidney disease (CKD) was defined as serum creatinine ≥ 1.5 mg/dL.

### 2.3. PCAT Definition

**Figure 1:**
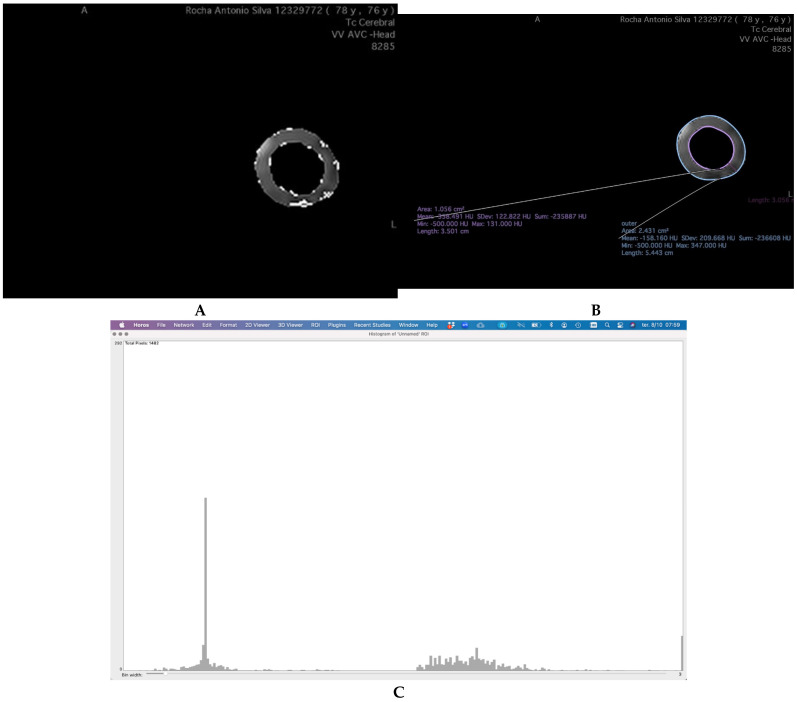
Workflow for PCAT ROI Definition and Attenuation Analysis in Carotid CT Angiography. (**A**) Demonstration of the definition of PCAT ROI, creating, in the region of biggest stenosis, a circumference 1 mm distant from the vascular wall (inner—pink) and one 3 mm away from the first (outer—blue). Isolating it and analyzing the number of pixels with attenuation between the ranges of HU defined. (**B**) Isolating the ROI from the rest of TC and isolating the three intervals of HU values. (**C**) Obtaining the number of pixels found with attenuation values in each group.

The region of greatest stenosis in the internal carotid artery was identified on CTA images. The Region of Interest (ROI) was defined as the area surrounding the vascular wall, delineated using the Closed Polygon tool in Horos^®^ (Horos Project (open-source community)—distributed at horosproject.org, accessed on 1November 2021) by creating two concentric circumferences:

- An inner boundary, positioned 1 mm from the vessel wall to exclude the influence of vascular calcification, as described in previous studies [15]
- An outer boundary, positioned 3 mm from the inner circumference (Figure 1A).

This method ensures optimal capture of relevant perivascular adipose tissue, minimizing the inclusion of surrounding non-adipose structures.

The ROI was carefully isolated to analyze only the tissue within this defined perimeter. Adipose tissue was identified based on attenuation values between −190 and −30 Hounsfield Units (HU) [16].

To further characterize the composition of the PCAT, the adipose tissue was categorized into three groups based on HU ranges: −190 to −120 HU; −119 to −70 HU; −69 to −30 HU [17]. This stratification allows for a more nuanced analysis of PCAT characteristics, potentially reflecting distinct perivascular tissue phenotypes or inflammatory states.

### 2.4. Image Analysis

CTA images were independently analyzed by two blinded observers (DST and JRN) using Horos^®^. Interobserver reliability was assessed using the intraclass correlation coefficient (ICC).

To analyze the HU-based groups within the ROI, the carotid artery regions were segmented, and pixel values within each HU interval were isolated using Horos^®^ (Figure 1B,C).

For radiomic feature extraction, the PyRadiomics extension within 3DSlicer^®^ was employed (https://pyradiomics.readthedocs.io, accessed on 15 July 2025). PyRadiomics is user-friendly and flexible for quantitative radiomic analysis, as demonstrated in prior research [7,18,19].

### 2.5. Outcome Assessment

We assessed the relationship between PCAT Hounsfield Unit (HU) ranges, radiomic features extracted from carotid computed tomography angiography (CCTA), and systemic inflammatory markers in patients undergoing carotid endarterectomy (CEA). The primary outcome measure was the neutrophil-to-lymphocyte ratio (NLR), and the secondary outcome was the red cell distribution width-coefficient of variation (RDW-CV), both derived from preoperative blood samples and serving as surrogate biomarkers of systemic inflammation.

### 2.6. Reproducibility Study

A pilot study of 10 cases was performed to investigate reproducibility (DST and JRN). The PCAT of both arteries, at the slices corresponding to the region of greatest stenosis, was segmented twice with a three-month interval between sessions.

### 2.7. Statistical Analysis

Data collection and analysis were performed using IBM SPSS Statistics (IBM Corp., release 2023. IBM SPSS Statistics for Windows, version 29.0.2.0, Armonk, NY, USA). Normally distributed data are presented as mean and standard deviation (SD), non-normal with median and interquartile range, while categorical variables are expressed as frequency and corresponding percentage.

For univariable analysis, χ^2^ or Fisher’s exact test were used for qualitative variables, and Student’s *t*-test and Mann–Whitney-U were used for quantitative variables, according to their normality. A linear regression model was employed to assess the association between PCAT radiomics and inflammatory markers. The small sample size precluded a multivariate regression analysis to be conducted. A *p*-value <0.05 was considered statistically significant.

## 3. Results

The study included 20 patients with a mean age of 71.8 years (±6.9). Of these, 16 (75.0%) were male and 4 (25.0%) were female. Laterality was nearly balanced, with 10 (50%) patients involving the right side the mean value was used for the analysis.

Cardiovascular risk factors were prevalent among the study sample, with 18 (90.0%) having hypertension, 11 (45.0%) diabetes, 12 (60.0%) a history of smoking, and 19 (95.0%) dyslipidemia. Obesity and chronic kidney disease were present in 3 (15.0%) and 4 (20.0%) patients, respectively. Peripheral artery disease was noted in 7 (35.0%), coronary artery disease in 5 (25.0%), and chronic obstructive pulmonary disease in 2 (10.0%), while congestive heart failure was absent.

Most patients were asymptomatic (16; 80.0%), while 4 (20.0%) were symptomatic. One patient (5.0%) had a prior history of stroke. Regarding antiplatelet therapy, 11 (55.0%) were on acetylsalicylic acid (ASA) for more than two weeks, and 2 (10.0%) were on clopidogrel.

Hematological markers showed a mean NLR of 5.79 (±4.0) and RDW-CV of 13.75% (±1.09) (Table 1).

Preoperative imaging in 20 patients revealed an outer PCAT area of 178.98 ± 42.4 (right) and 185.41 ± 52.3 (left), inner PCAT area of 71.38 ± 33.3 (right) and 65.17 ± 27.00 (left), and total PCAT area of 108.27 ± 63.6 (right) and 120.24 ± 26.1 (left). Fat pixel counts were comparable between sides (66.77 ± 68.3 right, 63.94 ± 75.3 left).

Pixel intensity distribution across HU ranges were minimal in the highest range (−69 to −30 HU) and progressively increased in lower ranges (−190 to −120 HU). The mean gray value was 1.51 ± 0.65, providing a baseline for further analysis (Table 2).

Univariate linear regression analysis revealed significant associations between NLR and PCAT characteristics. The outer area was inversely associated with NLR (β = −0.677, t = 5.151, *p* < 0.001), indicating that larger outer areas of PCAT are linked to lower NLR values. Similarly, the pixel range between −190 and −120 demonstrated a strong negative association with NLR (β = −3.809, t = 7.281, *p* < 0.001), while the pixel range between −119 and −70 showed a strong positive correlation (β = 3.814, t = 7.495, *p* < 0.001). Although the contrast variable exhibited a negative trend (β = −0.422, t = −1.920), this association did not reach statistical significance (*p* = 0.072) (Table 3).

Univariate linear regression analysis for RDW-CV identified significant and non-significant associations with key PCAT variables. Uniformity was positively associated with RDW-CV (β = 0.494, t = 2.411, *p* = 0.027), indicating that higher uniformity correlates with increased RDW-CV. Conversely, contrast showed a negative trend (β = −0.402, t = −1.861), though this association did not achieve statistical significance (*p* = 0.079) (Table 4).

## 4. Discussion

The study reveals significant associations between pericarotid adipose tissue characteristics and inflammatory markers, particularly the NLR and RDW-CV. Larger outer areas of PCAT were inversely associated with NLR, suggesting a potential anti-inflammatory effect. Moreover, specific pixel intensity ranges in PCAT imaging showed strong correlations with NLR, indicating that PCAT composition may reflect systemic inflammatory status. Furthermore, uniformity in PCAT was positively associated with RDW-CV, a marker of cardiovascular risk.

The inverse relationship between the outer PCAT area and NLR is in line with previous research suggesting that healthy PCAT exerts anti-inflammatory effects. Functional PCAT produces anti-inflammatory adipokines such as adiponectin, which can suppress vascular inflammation [20]. This supports the concept of PCAT as an active endocrine organ, capable of modulating both vascular function and systemic inflammation [1]

The contrasting associations of different pixel intensity ranges with NLR may further highlight the complex composition of pericarotid adipose tissue. The pixel ranges from −190 to −120 HU showed a strong negative correlation with NLR, while the range from −119 to −70 HU exhibited a strong positive correlation. These divergent relationships suggest that specific structural or cellular features of PCAT—captured through variations in attenuation—are associated with certain inflammatory states [21]. The negative correlation observed with the −190 to −120 HU range may reflect the presence of healthier adipose tissue, potentially richer in brown adipocytes or with more favorable secretory profile [6,22]. Previous studies have demonstrated that PCAT attenuation, measured in HU, can serve as a non-invasive imaging biomarker of coronary inflammation and higher attenuation values are associated with increased inflammation [21,23].

PCAT is composed of various cell types, including adipocytes, immune cells, and stromal cells, each contributing to its function and inflammatory potential. Its composition can shift in response to local or systemic inflammatory signals, which may in turn alter its CT attenuation. The browning of PCAT, characterized by increased thermogenic activity, has been associated with improved vascular health, potentially explaining the observed inverse relationship between certain attenuation ranges and systemic inflammation [6].

The positive association between PCAT uniformity and RDW-CV (β = 0.494, *p* = 0.027) is noteworthy, suggesting that structural or compositional changes in PCAT may associate with erythrocyte variability, possibly through shared inflammatory pathways. This supports the growing interest in Perivascular adipose tissue (PVAT) imaging as a tool for assessing cardiovascular risk, consistent with previous findings linking PCAT inflammation to vascular pathologies [24].

Although not statistically significant, the negative trend observed with the contrast variable may suggest an association between PCAT heterogeneity and systemic inflammation. This implies that more homogeneous PCAT might be linked to lower NLR levels, though further research is needed to confirm this hypothesis [25].

This study has several limitations. The relatively small sample size may restrict the generalizability of our findings. Larger, multicenter studies are necessary for external validation. The ROI definition encompassed a larger area than previous studies, which may have included non-adipose tissues with similar HU values, potentially affecting the accuracy of PCAT quantification.

Additionally, the cross-sectional design precludes causal inference between PCAT characteristics and systemic inflammation. Longitudinal studies could better assess the temporal dynamics of PCAT composition and their relationship to postoperative outcomes. While radiomics-based analysis is a promising approach to assessing PCAT, variability in segmentation and manual analysis techniques may introduce observer bias. To improve reproducibility, efforts toward standardized imaging protocols and automated analysis tools are recommended.

Finally, potential confounding variables—including medication use, lifestyle factors, and genetic predisposition—were not accounted for. Incorporating these factors into future research could further refine the clinical utility of PCAT imaging as a predictive tool in vascular surgery.

These findings contribute to the growing body of evidence suggesting that PCAT is not a passive fat depot, but rather an active metabolic and endocrine tissue involved in vascular homeostasis and systemic inflammation regulation. This concept is further supported by studies on PVAT’s role in microcirculatory modulation, as well as in conditions like hypertension and obesity.

The ability to detect these nuanced relationships through non-invasive imaging techniques highlights the potential of PVAT analysis as a tool for assessing cardiovascular risk and vascular health. Future research should prioritize longitudinal studies to establish the temporal relationship between changes in PVAT characteristics and systemic inflammation. Furthermore, combining PVAT imaging with other biomarkers and functional vascular assessments could offer a more comprehensive understanding of PVAT’s role in cardiovascular health and disease. This integrated approach is supported by studies demonstrating the anti-inflammatory role of factors such as ghrelin in the progression of atherosclerosis [26].

## 5. Conclusions

In conclusion, this study provides evidence for the intricate relationship between PCAT characteristics and systemic inflammation. These findings support the growing recognition of PCAT as a critical component in vascular health and disease, emphasizing its potential as both a diagnostic marker and therapeutic target in cardiovascular medicine. As our understanding of PCAT biology expands, we stand at the cusp of a new era in cardiovascular imaging and risk assessment, with PVAT analysis poised to play a central role in the development of personalized medicine strategies for the prevention and treatment of cardiovascular disease.

## Figures and Tables

**Table 1:** Demographics, comorbidities and binary analysis.

	Total (n = 20)
Gender, n (%)	
Male	16 (75.0)
Female	4 (25.0)
Age (years)	
Mean (±SD)	71.8 (±6.9)
Side	
Right, n (%)	10 (50.0)
Left, n (%)	10 (50.0)
CV risk-factors	
Hypertension, n (%)	18 (90.0)
Diabetes, n (%)	11 (45.0)
Smoking history, n (%)	12 (60.0)
Dyslipidemia, n (%)	19 (95.0)
Obesity, n (%)	3 (15.0)
CKD, n (%)	4 (20.0)
PAD, n (%)	7 (35.0)
CAD, n (%)	5 (25.0)
COPD, n (%)	2 (10.0)
CHF	0 (0.0)
AF, n (%)	2 (10.0)
ASA, n (%)	
II	1 (5.0)
III	18 (90.0)
IV	1 (5.0)
Asymptomatic, n (%)	16 (80.0)
Symptomatic, n (%)	4 (20.0)
TIA	0 (0.0)
Stroke	1 (5.0)
Stenosis degree	85.0 ± 10.8
Mean (±SD) (%)	
Contralateral stenosis degree	
Mean (±SD) (%)	56 ± 7.0
Contralateral stenosis (>50%)	5 (25)
AAS > 2 weeks, n (%)	11 (55.0)
Clopidogrel, n (%)	2 (10.0)
NLR	5.79 (±4.0)
RDW-CV (%)	13.75 (±1.09)

Abbreviations: AAS—Acetylsalicylic acid for more than two weeks before surgery; AF—atrial fibrillation; ASA—American Society of Anesthesiologists Physical Status Classification System; CAD—coronary artery disease; CHF—cardiac heart failure; CKD—chronic kidney disease (creatinine 1.5 mg/dL); COPD—chronic obstructive pulmonary disease; CV—cardiovascular; Obesity—body mass index > 30 kg/m^2^; NLR—Neutrophil to Lymphocyte ratio; PAD—peripheral artery disease; RDW-CV—red blood cell with—coefficient of variation; SD—standard deviation; TIA—transient ischemic attack.

**Table 2:** Preoperative radiologic parameters.

	Total (n = 20)
AreaRightOutter	178.98 ± 42.4
AreaRightInner	71.38 ± 33.3
AreaRightTotal	108.27 ± 63.6
FatPixelsRight	66.77 ± 68.3
pixel190to120R	0.05 ± 0.22
pixel119to70R	18.45 ± 35.4
pixel69to30R	48.2 ± 37.3
AreaLeftOutter	185.41 ± 52.3
AreaLeftInner	65.17 ± 27.00
AreLeft	120.24 ± 26.1
FatpixelsLeft	63.94 ± 75.3
pixel190to120L	0.64 ± 2.40
pixel119to70L	18.29 ± 18.5
pixel69to30L	65.93 ± 60.8
Grayweale	1.51 ± 0.65

**Table 3:** Univariate linear regression for Neutrophile to Lymphocyte Ratio.

	Beta	t	*p*
Area Outer	−0.677	5.151	˂0.001
pixel190to120	−3.809	7.281	˂0.001
pixel119to70	3.814	7.495	˂0.001
Contrast	−0.422	−1.920	0.072

Beta—standardized regression coefficient. t—t-statistic. *p* = *p*-value.

**Table 4:** Univariate linear regression for RDW-CV.

	Beta	t	*p*
Uniformity	0.494	2.411	0.027
Contrast	−0.402	−1.861	0.079

Beta—standardized regression coefficient. t—t-statistic. *p* = *p*-value.

## Data Availability

The datasets used and/or analysed during the current study are available from the corresponding author on reasonable request.

## Abbreviations

Glossary	
CEA	carotid endarterectomy.
CT	computed tomography.
CTA	computed tomography angiography.
HU	Hounsfield units.
MRI	magnetic resonance imaging.
NLR	neutrophil-to-lymphocyte ratio.
PCAT	pericarotid adipose tissue.
RA	regional anesthesia.
RDW	red cell distribution width.
ROI	region of interest.

## References

[1] Chen Y., Qin Z., Wang Y., Li X., Zheng Y., Liu Y. (2021). Role of Inflammation in Vascular Disease-Related Perivascular Adipose Tissue Dysfunction. Front. Endocrinol..

[2] Pereira-Neves A., Rocha-Neves J., Fragão-Marques M., Duarte-Gamas L., Jácome F., Coelho A., Cerqueira A., Andrade J.P., Mansilha A. (2021). Red blood cell distribution width is associated with hypoperfusion in carotid endarterectomy under regional anesthesia. Surgery.

[3] Duarte-Gamas L., Pereira-Neves A., Jácome F., Fragão-Marques M., Vaz R.P., Andrade J.P., Rocha-Neves J.P. (2020). Red Blood Cell Distribution Width as a 5-Year Prognostic Marker in Patients Submitted to Carotid Endarterectomy. Cerebrovasc. Dis. Extra.

[4] Yu Y., Cui W.H., Cheng C., Lu Y., Zhang Q., Han R.Q. (2021). Association between neutrophil-to-lymphocyte ratio and major postoperative complications after carotid endarterectomy: A retrospective cohort study. World J. Clin. Cases.

[5] Liu X., Wu F., Jia X., Qiao H., Liu Y., Yang X., Li Y., Zhang M., Yang Q. (2023). Pericarotid adipose tissue computed tomography attenuation distinguishes different stages of carotid atherosclerotic disease: A cross-sectional study. Quant. Imaging Med. Surg..

[6] Cai M., Zhao D., Han X., Han S., Zhang W., Zang Z., Gai C., Rong R., Gao T. (2023). The role of perivascular adipose tissue-secreted adipocytokines in cardiovascular disease. Front. Immunol..

[7] Nie J.Y., Chen W.X., Zhu Z., Zhang M.Y., Zheng Y.J., Wu Q.D. (2024). Initial experience with radiomics of carotid perivascular adipose tissue in identifying symptomatic plaque. Front. Neurol..

[8] Le E.P.V., Rundo L., Tarkin J.M., Evans N.R., Chowdhury M.M., Coughlin P.A., Pavey H., Wall C., Zaccagna F., Gallagher F.A. (2021). Assessing robustness of carotid artery CT angiography radiomics in the identification of culprit lesions in cerebrovascular events. Sci. Rep..

[9] AlSheikh S., Aljabri B., Alanezi T., Al-Salman M., Aldossary M.Y., Almashat A.H., Elmutawi H.S., Aldoghmani R.A., Altuwaijri T., Iqbal K. (2024). Outcomes of carotid endarterectomy: Insights from a single-center retrospective cohort study. Saudi Med. J..

[10] Mazzotta C., Basu S., Gower A.C., Karki S., Farb M.G., Sroczynski E., Zizza E., Sarhan A., Pande A.N., Walsh K. (2021). Perivascular Adipose Tissue Inflammation in Ischemic Heart Disease. Arter. Thromb. Vasc. Biol..

[11] Goncalves V.A., Geiger M.A., Sarti D.A., Guillaumon A.T. (2023). Association between platelet lymphocyte ratio and neutrophil lymphocyte ratio and clinical outcomes following carotid endarterectomy. J. Vasc. Bras..

[12] von Elm E., Altman D.G., Egger M., Pocock S.J., Gøtzsche P.C., Vandenbroucke J.P., Strobe Initiative (2014). The Strengthening the Reporting of Observational Studies in Epidemiology (STROBE) Statement: Guidelines for reporting observational studies. Int. J. Surg..

[13] Naylor R., Rantner B., Ancetti S., de Borst G.J., De Carlo M., Halliday A., Kakkos S.K., Markus H.S., McCabe D.J., Sillesen H. (2023). Editor’s Choice—European Society for Vascular Surgery (ESVS) 2023 Clinical Practice Guidelines on the Management of Atherosclerotic Carotid and Vertebral Artery Disease. Eur. J. Vasc. Endovasc. Surg..

[14] Oates C.P., Naylor A.R., Hartshorne T., Charles S.M., Fail T., Humphries K., Aslam M., Khodabakhsh P. (2009). Joint recommendations for reporting carotid ultrasound investigations in the United Kingdom. Eur. J. Vasc. Endovasc. Surg..

[15] Yu M., Meng Y., Zhang H., Wang W., Qiu S., Wang B., Bao Y., Du B., Zhu S., Ge Y. (2022). Associations between pericarotid fat density and image-based risk characteristics of carotid plaque. Eur. J. Radiol..

[16] Lan Y., Shang J., Ma Y., Zhen Y., Dang Y., Ren D., Liu T., Ju R., Guo N., Wang X. (2024). A new predictor of coronary artery disease in acute ischemic stroke or transient ischemic attack patients: Pericarotid fat density. Eur. Radiol..

[17] Tan N., Dey D., Marwick T.H., Nerlekar N. (2023). Pericoronary Adipose Tissue as a Marker of Cardiovascular Risk: JACC Review Topic of the Week. J. Am. Coll Cardiol..

[18] Polidori T., De Santis D., Rucci C., Tremamunno G., Piccinni G., Pugliese L., Zerunian M., Guido G., Pucciarelli F., Bracci B. (2023). Radiomics applications in cardiac imaging: A comprehensive review. Radiol. Med..

[19] van Griethuysen J.J.M., Fedorov A., Parmar C., Hosny A., Aucoin N., Narayan V., Beets-Tan R.G.H., Fillion-Robin J.C., Pieper S., Aerts H.J.W.L. (2017). Computational Radiomics System to Decode the Radiographic Phenotype. Cancer Res..

[20] Tong Y., Zuo Z., Li X., Li M., Wang Z., Guo X., Wang X., Sun Y., Chen D., Zhang Z. (2023). Protective role of perivascular adipose tissue in the cardiovascular system. Front. Endocrinol..

[21] Ma R., Fari R., Van Der Harst P., N. De Cecco C., E. Stillman A., Vliegenthart R., Van Assen M. (2023). Evaluation of pericoronary adipose tissue attenuation on CT. Br. J. Radiol..

[22] Madaelil T.P., Sharma A., Hildebolt C., Parsons M. (2018). Using Correlative Properties of Neighboring Pixels to Improve Gray-White Differentiation in Pediatric Head CT Images. AJNR Am. J. Neuroradiol..

[23] Butt K., D’SOuza J., Yuan C., Jayakumaran J., Nguyen M., I Butt H., Abusaada K. (2020). Correlation of the Neutrophil-to-Lymphocyte Ratio (NLR) and Platelet-to-Lymphocyte Ratio (PLR) with Contrast-Induced Nephropathy in Patients With Acute Coronary Syndrome Undergoing Percutaneous Coronary Interventions. Cureus.

[24] Adachi Y., Ueda K., Takimoto E. (2023). Perivascular adipose tissue in vascular pathologies—A novel therapeutic target for atherosclerotic disease?. Front. Cardiovasc. Med..

[25] Rami A.Z.A., Hamid A.A., Anuar N.N.M., Aminuddin A., Ugusman A. (2022). Exploring the Relationship of Perivascular Adipose Tissue Inflammation and the Development of Vascular Pathologies. Mediat. Inflamm..

[26] Adachi Y., Ueda K., Nomura S., Ito K., Katoh M., Katagiri M., Yamada S., Hashimoto M., Zhai B., Numata G. (2022). Beiging of perivascular adipose tissue regulates its inflammation and vascular remodeling. Nat. Commun..

